# Exome sequencing can misread high variant allele fraction of somatic variants in *UBA1* as hemizygous in VEXAS syndrome: a case report

**DOI:** 10.1186/s41927-022-00281-z

**Published:** 2022-08-30

**Authors:** Matheus V. M. B. Wilke, Eva Morava-Kozicz, Matthew J. Koster, Christopher T. Schmitz, Shannon Kaye Foster, Mrinal Patnaik, Kenneth J. Warrington, Eric W. Klee, Filippo Pinto e Vairo

**Affiliations:** 1 Center for Individualized Medicine, Mayo Clinic, Rochester, MN 55905 USA; 2 Department of Clinical Genomics, Mayo Clinic, 200 First St SW, Rochester, MN 55905 USA; 3 Department of Laboratory Medicine and Pathology, Mayo Clinic, Rochester, MN 55905 USA; 4 Division of Rheumatology, Department of Medicine, Mayo Clinic, Rochester, MN 55905 USA; 5 Division of Dermatology, Department of Medicine, Mayo Clinic, Rochester, MN 55905 USA; 6 Division of Hematology, Department of Medicine, Mayo Clinic, Rochester, MN 55905 USA; 7 Department of Quantitative Health Sciences, Mayo Clinic, Rochester, MN 55905 USA

**Keywords:** VEXAS syndrome, Variant allele frequency, X-Linked spinal muscular atrophy 2, Case report

## Abstract

**Background:**

VEXAS syndrome (vacuoles, E1 enzyme, X-linked, autoinflammatory, somatic syndrome) is a recently described syndrome caused by a somatic missense variant at the methionine-41 (p.(Met41)) position in the ubiquitin-like modifier activating enzyme 1 (*UBA1*) in Xp11.3. Germline pathogenic variants in *UBA1* are associated with a distinct phenotype: a syndrome with severe neurologic features associated with loss of anterior horn cells and infantile death denominated X-Linked Spinal Muscular Atrophy 2 (SMAX2) (OMIM 301,830).

**Case presentation:**

We report a male individual with the phenotype of VEXAS syndrome that was initially identified through exome sequencing (ES) as having a hemizygous germline variant in *UBA1* due to high variant allele frequency (VAF). Research Sanger sequencing was able to confirm the absence of the p.(Met41Val) variant in a skin biopsy and in gastric mucosa tissue sample confirming the variant happened as a postzygotic event.

**Conclusions:**

The present case exemplifies the diagnostic challenge that was imposed by the high VAF detected by ES that failed to correctly demonstrate that the variant was in a mosaic state. Sequencing of different tissues should be considered when there is conflict between the *UBA1* variant status and the clinical findings.

**Supplementary Information:**

The online version contains supplementary material available at 10.1186/s41927-022-00281-z.

## Key message

- Exome sequencing can misread high variant allele fraction in *UBA1* as hemizygous in VEXAS syndrome.

## Background

VEXAS syndrome (vacuoles, E1 enzyme, X linked, autoinflammatory, somatic syndrome) is a recently described syndrome caused by a somatic missense variant at the methionine-41 (p.(Met41)) position in the ubiquitin-like modifier activating enzyme 1 (*UBA1*) in Xp11.3. Ubiquitylation is an important post-translation biological process for hematopoiesis and the regulation of almost all host cellular processes, including host–pathogen interactions, inflammatory signaling, phagosomal maturation, autophagy, and apoptosis [1, 2].

All cases described in the literature corroborate that *UBA1* variants associated with VEXAS syndrome are found in somatic cells and affects predominantly males, with only 4 female cases reported, all related to monosomy X [3–7]. The main symptoms of VEXAS syndrome include rheumatologic manifestations such as arthralgias, ear and nose chondritis, recurrent fevers, dermatologic conditions, and pulmonary inflammatory manifestations [3–5].

Germline pathogenic variants in *UBA1* are associated with a distinct phenotype: a syndrome with severe neurologic features associated with loss of anterior horn cells and infantile death denominated X-Linked Spinal Muscular Atrophy 2 (SMAX2) (OMIM 301,830).

We report on an individual with the phenotype of VEXAS syndrome initially identified as having an apparent hemizygous germline variant in *UBA1* due to high variant allele frequency (VAF) in a clinical exome sequencing (ES)*.*

## Case presentation

A 46-year-old previously healthy male presented with priapism, chronic recurrent fevers, and elevated inflammatory markers treated with intermittent glucocorticoids. There was no other significant family history of autoimmune disorders or similar symptoms.

**Fig. 1:**
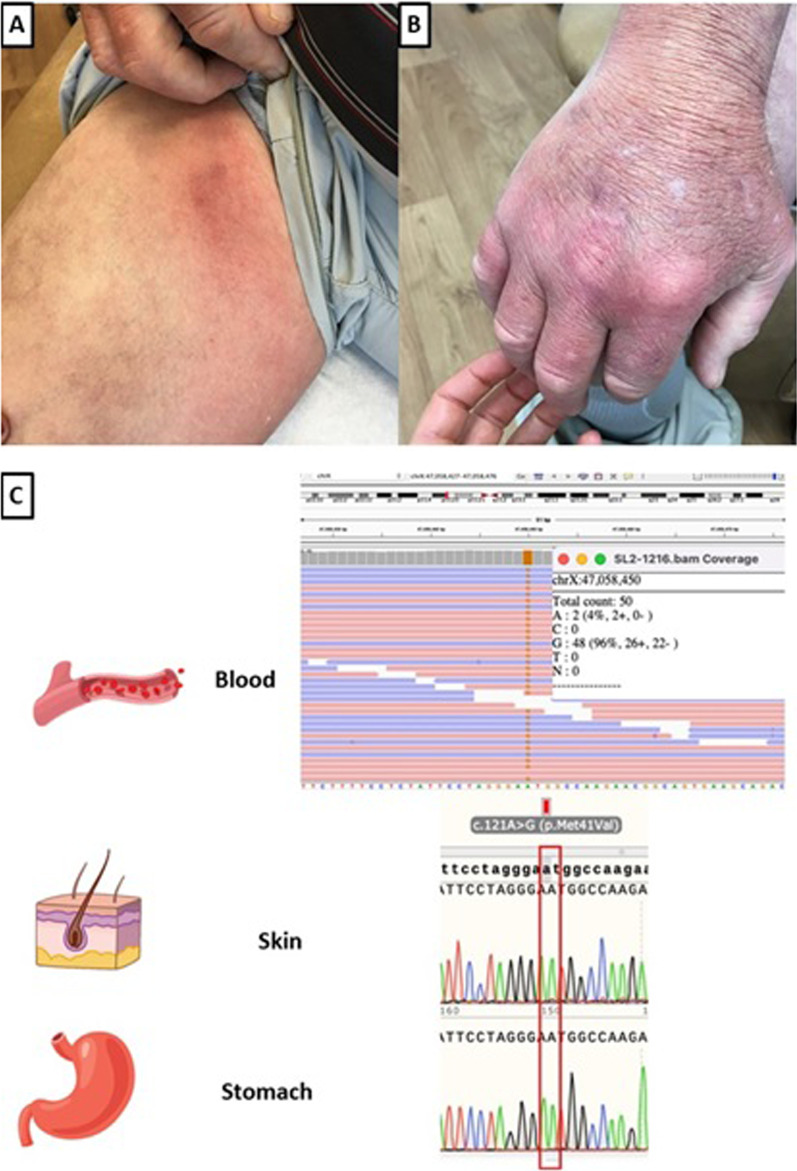
VEXAS syndrome skin lesions presented by the patient and Integrative Genome Viewer (IGV) and Sanger-sequencing electropherogram. **A** Edema and erythema with few subcutaneous indurated nodules in a linear, somewhat ropey pattern on right inner thigh. **B** Right dorsal hand is edematous and erythematous. Biopsy of the lesion showed subcutaneous panniculus with lipocyte necrosis, lipomembranous change, and clusters of neutrophils in the panniculus. **C** The *UBA1* variant (NM_003334.3) c.121A > G: p.(Met41Val) is found in 96% of the reads in the blood. Sanger-sequencing in skin and stomach demonstrated the absence of the variant in these tissues

At the age of 55 years, he developed bilateral episcleritis and testicular pain. Two skin nodule biopsies were obtained, one showed panniculitis (Fig. 1A) and the other medium-vessel vasculitis. His laboratory findings included positive antinuclear antibody (ANA), positive anti-Ro (SS-A) and markedly elevated IL-2R (Table 1). The patient was clinically suspected to have polyarteritis nodosa (PAN) due to the presence of medium vessel vasculitis, reported in a skin biopsy, associated with testicular pain and swelling, or cryopyrin-associated periodic syndrome (CAPS). A multi-gene panel was performed with no pathogenic variants identified in 73 genes associated with monogenic autoimmune diseases.

**Table 1:** Serum laboratory investigation

Exam	Values	Reference range
*Hematology*		
Hemoglobin (g/dL)	**8.5**	13.2–16.6
Hematocrit (%)	**27.5**	38.3–48.6
Erythrocytes (× 10(12)/L)	**2.01**	4.35–5.65
MCV (fL)	**136.8**	78.2–97.9
RBC distrib width (%)	**18.8**	11.8–14.5
Platelet count (× 10(9)/L)	**48**	135–317
White blood cell count (× 10(9)/L)	8.5	3.4–9.6
*Inflammatory markers*		
C-reactive protein (MG/DL)	**11.6**	0.0–0.9
Lactate dehydrogenase (U/L)	**230**	122–222
Erythrocyte sedimentation rate (mm/hr)	**120**	0–15
*Autoimmune*		
Soluble interleukin-2 receptor level (unit/mL)	**3017**	45–1105
dsDNA Ab with reflex, IgG, S (IU/ML)	20.6	< 30
Antinuclear Ab, S (U)	**4.2**	≤ 1.0
Cyclic citrullinated peptide Ab, S (U)	< 15.6	< 15.0
Centromere Ab, IgG, S (U)	< 0.2	< 1.0
SS-A/Ro Ab, IgG, S (U)	**7.9**	< 1.0
SS-B/La Ab, IgG, S (U)	< 0.2	< 1.0
Sm Ab, IgG, S(U)	< 0.2	< 1.0
RNP Ab, IgG, S(U)	< 0.2	< 1.0
Scl 70 Ab, IgG, S(U)	< 0.2	< 1.0
Jo 1 Ab, IgG, S (U)	< 0.2	< 1.0
Rheumatoid factor (IU/ML)	< 15	< 15
Ribosome P Ab, IgG, S (U)	< 0.2	< 1.0
Myeloperoxidase Ab, S (U)	< 0.2	< 0.4
Proteinase 3 Ab (PR3) (U):	< 0.2	< 0.4
*Complement*		
Complement, total, S (U/ML)	69	30–75
C1 esterase inhib, functional, QN (%)	** > 90**	> 67
Complement C1q, S (MG/DL)	22	12–22
Complement C4, S (MG/DL)	24	14–40
C1 esterase inhibitor antigen, S (MG/DL)	36	19–37
Complement, total, S (U/ML)	69	30–75
*Immunoglobulins*		
Haptoglobin (MG/DL)	**228**	30–200
Immunoglobulin A (IgA) (MG/DL)	157	61–356
Immunoglobulin E (IgE) (KU/L)	3.4	≤ 214
Immunoglobulin G (IgG) (MG/DL)	976	767–1590
Immunoglobulin M (IgM) (MG/DL)	47	37–286
Immunoglobulin Subclass IgG4 (MG/DL)	9.3	2.4–121
*Miscellaneous*		
Carcinoembryonic Ag (CEA) (NG/ML)	1.2	
Carbohydrate Ag 19–9, S (U/ML)**	**1288**	< 35
Methylmalonic acid, quantitative (NMOL/ML)	0.26	≤ 0.40
PNH RBC-partial Ag loss (%)	0.0	0.00–0.99
PNH RBC-complete Ag loss (%)	0.0	0.00–0.01

Abnormal results are shown in bold.

** The Ca19-9 was ordered to further evaluate pancreatic cysts found in his abdominal CT scam in the previous year. Whole body PET was normal

The patient received chronic prednisone (average dosage 20 mg/day) to control inflammatory symptoms. Adverse effects of long-term use of glucocorticoids included type 2 diabetes and cataracts noted around the age of 60 years. He also developed symmetric bilateral high-tone sensorineural hearing loss, which at the time was presumed due to use of Amikacin to treat chronic, recurrent disseminated *Mycobacterium chelonae.* Sequential trials of different medications were unable to successfully control the symptoms or allow successful glucocorticoid tapering (Additional file 1: Table S1). He was trialed on intravenous immunoglobulin therapy by the age of 60 which helped increase his blood counts and ameliorate symptoms.

Three bone marrow (BM) biopsies were performed between age 59 and 61 years. All three samples showed marked hypercellularity (highest 90%), with decreased erythropoiesis, increased granulopoiesis with mild cytologic atypia, and moderately decreased megakaryopoiesis. Cytoplasmic vacuolization was observed in both erythroid and myeloid precursors.

At 62 years of age, the patient was referred to the Department of Clinical Genomics because of ongoing undiagnosed autoinflammatory symptoms. After comprehensive genetic investigation, including ES, the patient remained undiagnosed. He died at age 63 due to sepsis without a unifying diagnosis.

Two years after initial ES and one year after the patient’s death, the clinical genetic laboratory issued a revised report noting a hemizygous (NM_003334.3) c.121 A>G-p.(Met41Val) variant in *UBA1* present in 96% of the reads (48/50 total reads). In light of the patient’s phenotype being discordant with known features of symptoms caused by germline *UBA1* variants, but concordant with recently reported auto-inflammatory disease related to somatic *UBA1* changes, additional testing was performed to elucidate the case. Postmortem research Sanger sequencing on archived tissue was able to confirm the absence of the p.(Met41Val) variant in a skin biopsy and in a gastric mucosa tissue sample both obtained premortem (Fig. 1B). The absence of the variant in these tissues determined the variant as a postzygotic event. This confirmed the somatic nature of the p.(Met41Val) variant in the blood DNA, allowing for a definitive diagnosis, and appropriate genetic counseling and familial risk assessment.

## Discussion and conclusion

Variants associated with VEXAS syndrome in p.(Met41) of *UBA1* result in reduction of functional cytoplasmic UBA1 resulting in upregulated cellular stress responses and activation of multiple innate immune pathways (such as elevated interferon-γ, TNF, IL-6, IL-8) causing multiorgan involvement as the symptoms progress [6]. The mean age of VEXAS onset is 67 years, ranging from 47 to 79 years with fever being reported as one of the most common symptoms [2] [7]. Fever in our patient was present only at the beginning of his investigation when CAPS was suspected. In a cohort of 16 patients with VEXAS syndrome, macrocytic anemia was found in all, lymphopenia in twelve individuals, and myelodysplastic syndrome in six individuals [4]. The clinical manifestations exhibited in VEXAS syndrome are notably variable. However, recent cluster analysis has identified a potential phenotype-genotype correlation between the type of *UBA1* variants and features exhibited [8]. The presented patient fits within the proposed phenotype of p.(Met41Val) with absence of chondritis, high CRP levels, and increased mortality risk.

VEXAS BM features are characterized by prominent cytoplasmic vacuoles in myeloid and erythroid cells. Even though these findings are not specific of VEXAS syndrome since they are present in other conditions such as myeloid neoplasms, copper deficiency, and alcohol intoxication, almost all of the confirmed cases in the literature have had this alteration [4, 9, 10]. Cytoplasmic vacuolation in hematopoietic cells was initially described for our patient but was not considered specific at the time, given it predated the clinical description of VEXAS syndrome by three years.

Many treatment-related complications have been described for patients with VEXAS syndrome including infections, cardiac involvement, stroke, and intestinal perforation indicating a poor prognosis with a mortality rate of 50% at the mean age of 76 [7, 11]. Our patient developed secondary complications such as cataracts, type 2 diabetes, and disseminated *Mycobacterium chelonei* infections, during the chronic use of corticosteroids. Sensorineural hearing loss was deemed associated with the use of amikacin, however this may have been due to VEXAS given it has been reported in 9% of the cases [2]. Inhibitors of TNF-alpha, IL-6, IL-1, and Janus Kinase (upregulated in VEXAS syndrome) have demonstrated varying success [2]. In one case series, no subject with VEXAS syndrome responded to disease-modifying anti-inflammatory drugs, while all were high-dose glucocorticoid-dependent [9]. Several immunosuppressive and immunomodulatory agents were used in our patient without adequate control. Stem-cell transplant has been proposed as potential treatment option and has shown preliminary favorable outcomes in case reports [10, 12–15]. A formal clinical trial evaluating allogeneic stem cell transplant in VEXAS is underway (ClinicalTrials.gov Identifier NCT05027945).

Germline variants in *UBA1* are associated with SMAX2 which is characterized by profound proximal hypotonia with muscle biopsy findings of neurogenic atrophy with no clinical overlap with VEXAS syndrome [16]. Germline p.(Met41Val) variant is not yet described to be associated with SMAX2. Sanger technique has been described as the methodology of choice to identify somatic variants in *UBA1* in the peripheral blood. Important to note, low-level mosaicism (< 20% of the cells) may be undetectable by Sanger sequencing so a next-generation technology with deeper coverage such as a targeted gene panel or ES might be warranted in some cases [3]. Interestingly, due to the high VAF, the *UBA1* pathogenic variant in our case was misinterpreted as being in a hemizygous state. High VAF has been described in the literature to occur in postzygotic event such as clonal hematopoiesis in variants in *TP53* with median VAF of 67.7% (range 46.9–84.7%) [17]. Regarding VEXAS, the highest value described in the literature was 83.35% in a patient diagnosed after analysis of peripheral-blood ES data [13]. Due to the absence of clinical features of SMAX2 in our patient, further testing in two different tissues samples through Sanger technique was used to confirm the somatic state of the *UBA1* variant. Testing to confirm the presence of somatic *UBA1* variant should be pursued, not only when the patient phenotype does not match that reported with SMAX2, but for patients with treatment-refractory inflammation with fever, cytopenia, cutaneous, and rheumatologic symptoms in general.

The present case exemplifies the diagnostic challenge that was imposed by the inaccurate recognition of the *UBA1* pathogenic variant as hemizygous on ES due to the VAF of the *UBA1* variant in the blood, which in this patient is the highest VAF recorded among VEXAS patients to date [3, 12]. It is important to consider sequencing different tissues when there is conflict between the *UBA1* variant status and the patient's clinical presentation. Even though treatment for VEXAS syndrome is still challenging, early diagnosis can be life-changing since there are reports of favorable outcomes after allogeneic stem cell transplant.

## Supplementary Information

**Additional file1 Table S1** Summary medication table outlining the patient’s medications used approximate durations and clinical response.

## Data Availability

The *UBA1* variant is a known pathogenic variant which is reported on ClinVar under accessions SCV001443136 and SCV002098354. If necessary, further information is available from the corresponding author on reasonable request. Further data sharing is not applicable to this article as no datasets were generated or analyzed during the current study.

## Abbreviations

BM: Bone marrow
CAPS: Cryopyrin-associated periodic syndrome
ES: Exome sequencing
PAN: Polyarteritis nodosa
SMAX2: X-linked spinal muscular atrophy 2
VAF: Variant allele frequency
VEXAS syndrome: Vacuoles, E1 enzyme, X linked, autoinflammatory, somatic syndrome
