# Supplementary material for: Exome sequencing can misread high variant allele fraction of somatic variants in UBA1 as hemizygous in VEXAS syndrome: a case report
Source: BMC Rheumatol. 2022 Aug 30;6:54. doi: 10.1186/s41927-022-00281-z (PMC9426024; doi:10.1186/s41927-022-00281-z)
Supplement: Supplementary file 1 — Additional file1 Table S1 Summary medication table outlining the patient’s medications used approximate durations and clinical response. [file 41927_2022_281_MOESM1_ESM.docx]

**Supplementary Table 1:** Summary medication table outlining the patients medications used approximate durations and clinical response.

| Medication | Dose/route/frequency | Duration | Response |
| --- | --- | --- | --- |
| Anakinra | 100 mg subQ, daily | 9 days | Stopped, severe skin reaction |
| Rilonacept | 320mg subQ x1 followed by 160 mg weekly | 3 months | PBR / NCR |
| Canakinumab | 150 mg subQ every 4 weeks | 3 months | PBR / NCR |
| Canakinumab | 300 mg subQ every 4 weeks | 3 months | PBR / NCR |
| Infliximab | 5 mg/kg, intravenously week 0,2,6 then every 6 weeks | 3 months | NBR / NCR |
| Infliximab | 10 mg/kg intravenously every 4 weeks | 3 months | NBR / NCR |
| Adalimumab | 40 mg subQ every 2 weeks | 3 months | NBR / NCR |
| Adalimumab | 40 mg subQ weekly | 2 months | NBR / NCR |
| Tocilizumab | 8 mg/kg, intravenous, monthly | 6 months | CBR / NCR |
| Secukinumab | 150 mg subQ weekly x 4 then monthly | 3 months | NBR / NCR |
| Secukinumab | 300 mg subQ monthly | 3 months | NBR / NCR |
| Tofacitinib | 11 mg oral, daily | 6 months | PBR / PCR |
| Rituximab | 1000 mg x 2, intravenously separated by 14 days | 6 months | NBR / NCR |
| Intravenous immunoglobulin | 1 g/kg, intravenously, x 2 consecutive days every 2 weeks for 2 months then monthly | 6 months | NBR / PCR, stopped due to insurance refusal to continue |
| Biologic response: *Complete biologic response* (CBR), full normalization of inflammatory markers; *Partial biologic response* (PBR), ≥ 50% reduction of C-reactive protein; *No biologic response* (NBR), less than 50% reduction of C-reactive protein  Clinical response: *Complete clinical response* (CCR), full resolution of symptoms; *Partial clinical response* (PCR), improvement in more than have of symptoms and/or reduction in symptom severity by ≥ 50%; *No clinical response* (NCR), less than 50% improvement in number of symptoms or symptom severity  *Given increase in steroids occurred with each flare/medication change, summary biologic and Clinical response assessed when prednisone dose lowered below 20 mg oral daily | | | |
